# Coping Strategies and Self-Efficacy in University Students: A Person-Centered Approach

**DOI:** 10.3389/fpsyg.2020.00841

**Published:** 2020-05-19

**Authors:** Carlos Freire, María del Mar Ferradás, Bibiana Regueiro, Susana Rodríguez, Antonio Valle, José Carlos Núñez

**Affiliations:** ^1^Department of Psychology, University of A Coruña, A Coruña, Spain; ^2^Department of Pedagogy and Didactics, University of Santiago de Compostela, Santiago de Compostela, Spain; ^3^Faculty of Psychology, University of Oviedo, Oviedo, Spain

**Keywords:** coping strategies, coping flexibility, stress, self-efficacy, university students

## Abstract

In daily academic life, students are exposed to a wide range of potentially stressful situations which could negatively affect their academic achievement and their health. Among the factors that could be weakened by academic stress, attention has been paid to expectations of self-efficacy, which are considered one of the most important determinants for student engagement, persistence, and academic success. From a proactive perspective, research on academic stress has emphasized the importance of coping strategies in preventing harmful consequences. In recent years, there has been a growing interest in discovering the extent to which individuals are able to combine different coping strategies and the adaptive consequences this flexibility entails. However, studies using this person-centered approach are still scarce in the academic context. On that basis, this current study had two objectives: (a) to examine the existence of different profiles of university students based on how they combined different approach coping strategies (positive reappraisal, support seeking, and planning) and (b) to determine the existence of differences in general expectations of self-efficacy between those coping profiles. A total of 1,072 university students participated in the study. The coping profiles were determined by latent profile analysis (LPA). The differences in the self-efficacy variable were determined using ANCOVA, with gender, university year, and degree type as covariates. Four approach coping profiles were identified: (a) low generalized use of approach coping strategies; (b) predominance of social approach coping approaches; (c) predominance of cognitive approach coping approaches; and (d) high generalized use of approach coping strategies. The profile showed that a greater combination of the three strategies was related to higher general self-efficacy expectations and vice versa. These results suggest that encouraging flexibility in coping strategies would help to improve university students’ self-efficacy.

## Introduction

The mental health of university students has been a growing concern in recent years (Milojevich and Lukowski, 2016). Various studies have demonstrated the high frequency of psychological symptoms associated with this stage of education (Blanco et al., 2008; Kim et al., 2015), with stress being one of the psychosocial problems that have become prevalent (Deasy et al., 2014; American College Health Association, 2018; Gustems-Carnicer et al., 2019). In their daily lives, university students have to face a wide variety of demands, both academic and non-academic, that could affect their well-being. Academic demands include adaptation to a new context, overwork, insufficient time to do their academic tasks, preparation for and doing of exams, and the pressure to perform (Beiter et al., 2015; Vizoso and Arias, 2016; Erschens et al., 2018; Webber et al., 2019). Non-academic demands include change of where they live; the need to create new social relationships; conflicts with partners, family, or friends; money worries; and concerns about future work (Howard et al., 2006; Galatzer-Levy et al., 2012; DeRosier et al., 2013; Beiter et al., 2015). Stress can bring with it significant harm to the student’s academic performance (e.g., reduced ability to pay attention or to memorize, less dedication to study, and more absences from class) (Chou et al., 2011; Turner et al., 2015), as well as to the student’s physical and psychological health (e.g., substance abuse, insomnia, anxiety, and physical and emotional exhaustion) (Waqas et al., 2015; Schönfeld et al., 2016). These harmful effects have triggered interest in the identification of individual psychological resources that could be protective factors against the inherent stressors of the university context (Tavolacci et al., 2013). These resources would modulate the relationship between the potential threats and the stress response, encouraging better psychological adjustment (Leiva-Bianchi et al., 2012). Two of the most widely studied resources are coping strategies and self-efficacy.

### Coping Strategies

Lazarus and Folkman (1984) thought of stress as an interactive process between the person and their surroundings, in which the influence of stressful events on physical and psychological well-being is determined by coping. From this widely accepted transactional approach, coping would come to be defined by cognitive and behavioral efforts employed in response to external or internal demands that the individual deems to be threats to their well-being.

Despite the documentation of more than 400 coping strategies (Skinner et al., 2003), they are generally categorized into two broad types (for a complete categorization, see Zimmer-Gembeck and Skinner, 2016): approach (also called active) strategies and evasive (or disengagement) strategies. Approach strategies involve cognitive and behavioral mechanisms aimed at making an active response to the stressor, directly changing the problem (primary control) or the negative emotions associated with it (secondary control). This category includes strategies such as planning, taking specific action, seeking support (instrumental and emotional), positive reappraisal of the situation, or acceptance. Evasive strategies are those which involve cognitive and behavioral mechanisms used to evade the stressful situation, such as distraction, denial, and wishful thinking. Based on this classification, there is a broad consensus that approach strategies are related to good academic, physical, and psychological adjustment (Clarke, 2006; Syed and Seiffge-Krenke, 2015; Gustems-Carnicer et al., 2019), whereas evasive strategies usually mean maladaptive consequences for the students (Tavolacci et al., 2013; Deasy et al., 2014; Skinner et al., 2016; Tran and Lumley, 2019).

### Self-Efficacy

Expectations of self-efficacy are a central element of the social cognitive theory proposed by Bandura (1997). This construct is about a person’s beliefs about their ability to mobilize courses of action needed to achieve desired personal goals. It is, therefore, a fundamental psychological resource for exercising control over events in one’s life (Wood and Bandura, 1989). In fact, self-efficacy is considered a powerful motivational, cognitive, and affective determinant of student behavior, with significant influence on their involvement, effort, persistence, self-regulation, and achievement (Schunk and Pajares, 2010; Honicke and Broadbent, 2016; Ritchie, 2016; Zumbrunn et al., 2019). These characteristics make self-efficacy an important variable in controlling stress (Bandura et al., 2003; Sahin and Çetin, 2017; Lanin et al., 2019), and it is a protection factor against the impact of day-to-day stressors at university (Freire et al., 2019; Schönfeld et al., 2019).

Although self-efficacy has commonly been characterized as an expectation that is strongly linked to a specific task or situation, various studies have demonstrated the existence of a more generalized belief—that is, general self-efficacy—around perceived competence in the face of a broad range of demands (Scholz et al., 2002; Feldman et al., 2015; Volz et al., 2019).

### Current Study

The literature reviewed reiterated the importance of considering both coping strategies and expectations of self-efficacy in protection against stress. However, far from being independent resources, some studies have suggested that coping strategies and self-efficacy are related. They postulate that coping behaviors would influence an individual’s expectations of control (Lazarus and Folkman, 1984), such that self-efficacy would be a mediator between coping strategies and the stress response (Zimmer-Gembeck and Skinner, 2016).

Given that, our study aimed to examine the possible influence of coping strategies on the expectations of self-efficacy in a population that is particularly vulnerable to stress, university students. Some studies have shown a positive, significant influence of approach coping strategies on self-efficacy in infant samples (Sandler et al., 2000) and in adults with rheumatoid arthritis (Keefe et al., 1997). However, as far as we are aware, there have been none in the university context.

The main contribution of this study lies in the analysis of student coping strategies using a person-centered focus. Traditionally, research on coping strategies has attempted to determine the suitability of a given strategy, evaluating the benefit or harm that it produces for the individual. This variable-centered approach assumes that certain coping mechanisms are universally adaptive or maladaptive, an argument that has been called the “fallacy of uniform efficacy” (Bonanno and Burton, 2013).

The very characterization of coping strategies as responses to a specific challenge demonstrates their situational specificity. This has led in recent years to the adoption of an approach based on the flexibility of coping, under the supposition that a single individual can combine different strategies, using one or the other depending on the specific situation they are facing (Eisenbarth, 2012; Kobylińska and Kusev, 2019). In this vein, the benefits provided by approach coping strategies are maximized if the individual employs problem-focused coping strategies (e.g., planning and seeking instrumental support) or emotion-centered strategies (e.g., positive reappraisal and seeking emotional support) based on the perceived controllability of the stressor facing them (Cheng, 2001; Siltanen et al., 2019). In contrast, people who are less flexible in their coping have a smaller repertoire of strategies, which are less effective adjusting to the specific demands of the situation (Cheng and Cheung, 2005).

Studying individuals’ profiles in light of the flexibility of their coping is therefore adopting a person-centered focus (Laursen and Hoff, 2006), making it possible to identify subgroups of students characterized by high internal similarity in their repertoire of coping strategies, who differ from the way that other students combine their strategies. An additional advantage over the traditional, variable-focused approaches is that studying profiles of flexibility of coping makes it possible to identify specific groups of individuals who can be prioritized in the design of interventions (Kaluza, 2000).

Considering a perspective based on coping flexibility, the research question we posed in this study was whether the different student profiles—in the way they combine their coping strategies—would be related to significantly different levels of general self-efficacy. In the university context, various studies have demonstrated that, in comparison to those with less flexible profiles, students who are more flexible in their coping demonstrate lower vulnerability to stress (Cheng, 2001; Kato, 2012; Doron et al., 2014; González Cabanach et al., 2018) and to depressive symptomatology (Gabrys et al., 2018; Hasselle et al., 2019), as well as greater psychological well-being (Freire et al., 2018). Based on that research, our hypothesis is that students who exhibit a more flexible profile of strategies will demonstrate significantly higher levels of self-efficacy than less flexible students.

Assuming that in the young population the use of approach coping strategies is more typical (Cheng et al., 2014), in our study, we examined coping profiles based on the combination of three approach strategies that are very common in educational contexts (Skinner et al., 2016): a primary control (planning), a secondary control (positive reappraisal), and a mixed type (seeking instrumental and emotional support). Similarly, given the extensive and varied range of demands faced by students in their daily lives (both academic and non-academic), we examined their level of general self-efficacy. Finally, in this study, we also tried to control for the effects of the variables gender, university year, and degree type. It would seem that men report higher levels of self-efficacy than women, with this difference emerging at the end of adolescence (Huang, 2013). It may also be the case that students in their first year of university, because of their inexperience, may have lower levels of self-efficacy than students with more academic experience (Honicke and Broadbent, 2016). As for the type of course, scientific disciplines have been related to lower levels of self-efficacy (Findley-Van Nostrand and Pollenz, 2017).

## Materials and Methods

### Participants

The study used a sample of 1,085 undergraduate students from the University of A Coruña (Spain). The inclusion criteria were for subjects to be undergraduate students at the time of the study. Exclusion criteria included failing to respond to more than 20% of the items. We excluded 13 cases because they failed to respond to enough items. There were a smaller number of missing values in 28 other cases, which were dealt with using full information maximum likelihood (FIML) via Mplus 7.11 (Muthén and Muthén, 1998–2012). This means that the definitive sample was made up of 1,072 students aged between 18 and 48 years (*M* = 21.09; *SD* = 3.16). Just over two thirds (*n* = 729; 68%) were women, and 343 (32%) were men. The distribution by degree course was as follows: 383 (37.5%) were studying educational sciences (infant education, primary education, social education, physical education, language and hearing, speech therapy, and educational psychology); 203 (19%) were studying health sciences (physiotherapy, nursing, and sports science); 207 (19.3%) were studying legal and social sciences (law and sociology); and 279 (26%) were studying technical sciences (architecture, technical architecture, and civil engineering). The distribution of students in terms of their university year was 304 (28.4%) in their first year, 307 (28.6%) in their second year, 302 (28.2%) in their third year, 91 (8.5%) in their fourth year, and 68 (6.3%) in their fifth year.

### Instruments

#### Coping Strategies

We used the coping scale from the Academic Stress Questionnaire to measure coping strategies (Cabanach et al., 2010). This instrument has 23 items evaluating three approach strategies for coping: positive reappraisal, support seeking, and planning. Positive reappraisal is a secondary control strategy in which the student seeks to reassign the stressful event, highlighting the positive (e.g., “When I am faced with a problematic situation, I forget unpleasant aspects and highlight the positive ones”). The psychometric properties were acceptable, in terms of both reliability (α = 0.860; ω = 0.864; construct reliability = 0.857; composite reliability = 0.857) and validity (convergent validity = 0.483; construct validity: χ^2^ = 119.87; *df* = 30; *p* > 0.05; *GFI* = 0.98; *AGFI* = 0.96; *TLI* = 0.96; *CFI* = 0.98; *RMR* = 0.03; *RMSEA* = 0.05). Support seeking is a mixed coping strategy, as the student can do that with the aim of seeking information and advice from others to resolve the issue at hand (e.g., “When I am faced with a problematic situation, I ask for advice from a family member or a close friend”) or they can seek consolation and emotional relief (e.g., “When I am faced with a problematic situation, I manifest my feelings and opinions to others”). The psychometric properties of this subscale were good, in reliability (α = 0.902; ω = 0.903; construct reliability = 0.900; composite reliability = 0.900) and validity (convergent validity = 0.566; construct validity: χ^2^ = 35.43; *df* = 12; *p* > 0.05; *GFI* = 0.99; *AGFI* = 0.98; *TLI* = 0.99; *CFI* = 0.99; *RMR* = 0.02; *RMSEA* = 0.04). Planning is a primary control strategy, characterized by analysis and the design of a plan of action aimed at resolving the problematic situation (“When I am faced with a problematic situation, I draw up an action plan and follow it”). The psychometric properties were acceptable, in terms of both reliability (α = 0.81; ω = 0.81; construct reliability = 0.85; composite reliability = 0.82) and validity (convergent validity = 0.504; construct validity: χ^2^ = 33.52; *df* = 8; *p* > 0.05; *GFI* = 0.99; *AGFI* = 0.97; *TLI* = 0.97; *CFI* = 0.98; *RMR* = 0.03; *RMSEA* = 0.05). The participants’ responses are recorded on a five-point Likert scale (1 = never to 5 = always).

#### Self-Efficacy

We used the Spanish validation of the General Self-efficacy Scale from Baessler and Schwarzer (1996). The scale has 10 items (e.g., “I can solve difficult problems if I try hard enough”) that the participants respond to on a Likert scale from 1 (never) to 5 (always). In this study, the psychometric properties were good, in reliability (α = 0.91; ω = 0.91; construct reliability = 0.909; composite reliability = 0.909) and validity (convergent validity = 0.514; construct validity: χ^2^ = 121.36; *df* = 30; *p* > 0.05; *GFI* = 0.98; *AGFI* = 0.96; *TLI* = 0.98; *CFI* = 0.98; *RMR* = 0.02; *RMSEA* = 0.05).

### Procedure

The study protocol was designed and executed in compliance with the code of ethics set out by the university in which the research was done, with the informed consent of all participants, as required by the Helsinki Declaration. Data collection was carried out at the beginning of the academic year in order to avoid periods of high academic demands (e.g., work overload and preparation for exams) that could favor greater emotional activation in students and, therefore, influence their responses to the questionnaires. Before beginning the study, the participants were informed of the objectives and were asked to participate; they were assured of anonymity and the confidentiality of their responses. Likewise, the instructor explained that students who did not wish to participate in the study could leave the classroom until the end of the tests, without any repercussions or negative consequences. The questionnaires were administered in the classrooms where the students had their usual classes, during normal class hours, and in a single session without a time limit.

### Data Analysis

To identify the student profiles according to the flexibility of their coping, we performed a latent profile analysis (LPA) (Lanza et al., 2003) using the statistical program Mplus 7.11 (Muthén and Muthén, 1998–2012). LPA allows the identification of latent categorical variables to group the subjects into classes (profiles), establishing what fits best from a finite set of models. The following were used as reference parameters to determine the optimum model: the Akaike Information Criterion (AIC), the Schwarz Bayesian information criterion (BIC), the BIC adjusted for sample size (SSA-BIC), the formal adjusted maximum likelihood ratio test from Lo et al. (2001) (LMRT), the parametric bootstrap likelihood ratio test (PBLRT), and the sample size for each subgroup. The AIC, BIC, and SSA-BIC indices are descriptive, the lowest values indicating the best fit of the model, whereas LMRT and PBLRT are the indices that allow the final decision to be made. The values of *p* associated with LMRT and PBLRT indicate whether the solution with more (*p* < 0.05) or fewer classes (*p* > 0.05) is the one with the best fit to the data. Another of the exclusion criteria was the existence of spurious classes (*n* ≤ 5% of the sample), which would indicate excessive extraction of profiles (Hipp and Bauer, 2006).

Once the optimal model was selected based on the above criteria, we moved on to determining its classifying accuracy using the entropy statistic and calculation of *a posteriori* probabilities as references. Another criterion for evaluating the validity of the model was a MANOVA analyzing the differences between classes in the three criterion variables (positive reappraisal, support seeking, and planning). Statistically significant differences between the three variables would indicate that the latent classes suggested by the model were distinct. Finally, the differences in self-efficacy between the different coping profiles were established using an ANCOVA, with gender, year, and degree type as covariables. The effect size of the differences between the groups was determined using partial eta squared and Cohen’s (1988) *d*: null, η_p_^2^ < 0.01 (*d* < 0.09); small, η_p_^2^ = 0.01 to η_p_^2^ = 0.058 (*d* = 0.10 to *d* = 0.49); medium, η_p_^2^ = 0.059 to η_p_^2^ = 0.137 (*d* = 0.50 to *d* = 0.79); and large, η_p_^2^ ≥ 0.138 (*d* ≥ 0.80). These analyses were performed using SPSS 26.0 (IBM Corp, 2019).

## Results

### Preliminary Analysis

Descriptive statistics and the values of (Pearson) correlations between the variables are given in Table 1. The asymmetry and kurtosis data indicate that the variables followed a normal distribution (all values between −1 and 1). Similarly, all of the correlations were statistically significant (*p* < 0.001). Statistically speaking, the results of the Bartlett sphericity test indicate that the variables were sufficiently intercorrelated [χ^2^(6) = 1,066.75; *p* < 0.001)], an important requirement for subsequent multivariate analysis.

**TABLE 1:** Means, standard deviations, and correlations for the three strategies for coping with stress and general self-efficacy (*N* = 1072).

	1	2	3	4
1. General self-efficacy				
2. Positive reappraisal	0.63			
3. Support seeking	0.21	0.22		
4. Planning	0.45	0.55	0.30	
M	3.34	3.01	3.44	3.05
SD	0.68	0.71	0.87	0.74
Skewness	−0.03	0.05	−0.15	0.07
Kurtosis	−0.44	−0.45	−0.79	−0.44

*All Pearson *r* correlation coefficients are significant at *p* < 0.001. General self-efficacy scale and coping strategies scale: 1 = never, 2 = sometimes, 3 = several times, 4 = many times, and 5 = always. Higher scores reflect greater levels of general self-efficacy and a higher use of coping strategies.*

### Identification of Coping Profiles

The fit of various latent profile models was examined (models from two to five classes). In the model fit, it was assumed that variances could differ between indicators within each group, with the restriction specifying that they be equal between the groups. Similarly, a restriction was set on the independence between indicators, both within and between groups.

Table 2 gives the results of the model fit. The analysis of fit was stopped at the five-class model for various reasons: (a) the values of BIC and SSA-BIC were higher in the five-class model than in the four-class model, and the AIC was almost the same in the two models; (b) the values of LMRT and PBLRT for the five-class model were not statistically significant (*p* > 0.05, in both cases), which indicated that the fit of this model was not better than that of the four-class model; (c) the five-class model included a group made up of fewer than 5% of the total sample, which indicated excessive extraction of profiles. In contrast, in the four-class model, all of the groups made up more than 5% of the total sample. Similarly, all of the data summarized in Table 2 indicated that the four-class model demonstrated better fit than the two- and three-class models, leading to the selection of the four-class model as the optimum.

**TABLE 2:** Statistics for the identification of fit of latent class models and classifying accuracy.

	Models of coping profiles			
	
	Two classes	Three classes	Four classes	Five classes
AIC	7, 045.953	6, 979.629	6, 947.676	6, 945.556
BIC	7, 095.726	7, 049.311	7, 037.267	7, 055.056
SSA-BIC	7, 063.964	7, 004.844	6, 980.096	6, 985.180
Entropy	0.638	0.607	0.639	0.705
Number of groups with *n* ≤ 5%	0	0	0	1
LMRT	397.586**	71.753*	38.571*	9.770
PBLRT	411.832**	74.324**	39.953**	10.120

The models were adjusted assuming that the variances could differ between the indicators within each group, but it was specified as a restriction that they be equal between groups. Likewise, independence between the indicators was imposed as a restriction, both within each group and between groups. AIC = Akaike information criterion; BIC = Schwarz Bayesian information criterion; SSA-BIC = BIC adjusted for the sample size; LMRT = adjusted Lo–Mendell–Rubin maximum likelihood ratio test; PBLRT = parametric bootstrap likelihood ratio test; *p < 0.01; **p < 0.001.

Table 3 gives the classifying accuracy of the four-class model, as well as the number of participants (overall sample and by gender) making up each class in that model, both in absolute terms (*n*) and as a percentage (%). The means associated with the groups the participants were assigned to are given in the main diagonal in the table in bold. The first group demonstrated a classification coefficient of 85%, whereas the other three groups had coefficients a little below 80%. Overall, these data indicate that the four-class model demonstrates adequate classification accuracy. Similarly, the value of the entropy statistic of this model (0.639) (Table 2), although modest, is acceptable (Nylund et al., 2007).

**TABLE 3:** Characterization of the latent profiles and classifying accuracy of the individuals in each profile.

	Latent profiles				*n* (%)	*n*_*gender*_ (%)	
			
	1	2	3	4		Female	Male
1. LACS	**0.848**	0.002	0.089	0.061	296 (27.61)	195 (65.9)	101 (34.1)
2. HACS	0.001	**0.796**	0.135	0.068	290 (27.05)	194 (66.9)	96 (33.1)
3. SAC	0.084	0.111	**0.770**	0.035	355 (33.12)	286 (80.6)	69 (19.4)
4. CAC	0.089	0.094	0.049	**0.768**	131 (12.22)	54 (41.2)	77 (58.8)

*LACS, profile of low approach coping strategies; HACS, profile of high approach coping strategies; SAC, profile with a prevalence of social approach coping strategies; CAC, profile with a prevalence of cognitive approach coping strategies. The coefficients associated with the groups to which the participants have been assigned are shown in bold.*

As an additional criterion for assessing the suitability of the four-class model, the results of the MANOVA showed statistically significant differences between the four classes in the three criterion variables: positive reappraisal [*F*(3, 1068) = 391.49; *p* < 0.001; η_p_^2^ = 0.524], support seeking [*F*(3, 1068) = 770.37; *p* < 0.001; η_p_^2^ = 0.684], and planning [*F*(3, 1068) = 463.61; *p* < 0.001; η_p_^2^ = 0.566]. The effect size was large in all cases.

### Description of Coping Profiles

**FIGURE 1:**
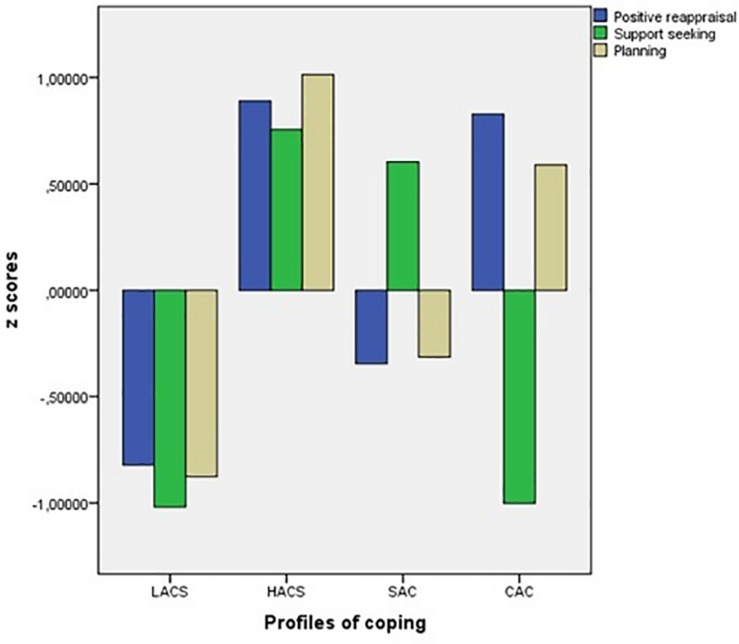
Graphical representation of coping profiles (standardized scores). LACS: profile of low approach coping strategies; HACS: profile of high approach coping strategies; SAC: profile with a prevalence of social approach coping strategies; CAC: profile with a prevalence of cognitive approach coping strategies.

The mean scores (direct and standardized) of the members of each of the latent classes (coping profiles) in the selected model are given in Table 4. The same profiles are shown graphically in Figure 1.

**TABLE 4:** Description of latent profiles (means, standard errors, and confidence intervals).

			Confidence intervals	
			
	*M*	*SE*	Lower 5%	Upper 5%
*LACS (n* = *296)*				
Positive reappraisal	2.45 (−0.82)	0.06	2.35	2.55
Support seeking	2.61 (−1.02)	0.05	2.53	2.69
Planning	2.42 (−0.88)	0.06	2.33	2.52
*HACS (n* = *290)*				
Positive reappraisal	3.61 (0.89)	0.07	3.49	3.73
Support seeking	4.07 (0.75)	0.05	3.99	4.16
Planning	3.72 (1.01)	0.06	3.62	3.83
*SAC (n* = *355)*				
Positive reappraisal	2.79 (−0.35)	0.06	2.69	2.88
Support seeking	3.95 (0.60)	0.06	3.86	4.04
Planning	2.85 (−0.31)	0.07	2.73	2.97
*CAC (n* = *131)*				
Positive reappraisal	3.52 (0.83)	0.10	3.35	3.69
Support seeking	2.71 (−1.00)	0.08	2.58	2.84
Planning	3.43 (0.59)	0.11	3.24	3.61

*LACS, profile of low approach coping strategies; HACS, profile of high approach coping strategies; SAC, profile with a prevalence of social approach coping strategies; CAC, profile with a prevalence of cognitive approach coping strategies. All measurement scales ranged from 1 to 5, where the highest scores reflect a higher level of approach coping strategies. Normalized mean scores are given in brackets (*z*).*

The first group (*n* = 296; 27.61%) was made up of students with low scores in the three approach coping strategies (profile of low approach coping strategies, LACS), who demonstrated low flexibility in the use of these strategies. The second group (*n* = 290; 27.05%) demonstrated the opposite, scoring highly in the three coping strategies (profile of high approach coping strategies, HACS). Compared to the other profiles, these were the students who demonstrated the most flexibility in deploying approach coping strategies. The third group was the largest (*n* = 355; 33.12%) and was made up of students with high scores in support seeking and low scores in positive reappraisal and planning. Given the overwhelmingly social nature of support seeking, we called this the social approach coping (SAC) profile. Finally, the smallest group in quantitative terms (*n* = 131; 12.22%) was made up of students demonstrating the opposite pattern to SAC, high scores in positive reappraisal and planning and low scores in support seeking. We called this the cognitive approach coping (CAC) profile as these students seemed to prefer more cognitive approach strategies, rather than social strategies.

### Relationship Between Coping Profiles and Self-Efficacy

Once the effects of gender, year, and degree course had been controlled for, the results of the ANCOVA demonstrated statistically significant differences between the coping profiles in the variable self-efficacy [*F*(3, 1065) = 140.638, *p* < 0.001, η_p_^2^ = 0.284), with a large effect size. The *a posteriori* tests (Scheffé) showed that the HACS profile scored highest in self efficacy, with statistically significant differences between it and the SAC and LACS profiles, the effect size being large in both cases (*d* = 0.98 and *d* = 1.55, respectively). The CAC profile also had significantly higher scores in self-efficacy than the SAC and LACS profiles, with large effect sizes (*d* = 0.88 and *d* = 1.46, respectively). The self-efficacy scores from the SAC profile were significantly higher than those from the LACS profile, with a medium effect size (*d* = 0.58). These data indicate that the LACS profile scored significantly lower in self-efficacy than the other coping profiles identified in this study. Table 5 gives the descriptive statistics for the four coping profiles with respect to the self-efficacy variable. When we looked at the covariables, there was no statistically significant effect found with the year variable, but there was with the degree type [*F*(1065) = 5.163, *p* < 0.05, η_p_^2^ = 0.005] and gender [*F*(1065) = 50.405, *p* < 0.001, η_p_^2^ = 0.045], although the effect size was null for the degree type and small for gender. Having noted the small effect of gender on self-efficacy, we looked more deeply at this interaction in each of the coping profiles. In the LACS [*t*(294) = 6.56, *p* < 0.001, *d* = 0.45], HACS [*t*(288) = 4.17, *p* < 0.001, *d* = 0.27], and SAC profiles [*t*(353) = 3.43, *p* < 0.01, *d* = 0.26], men scored significantly higher in self-efficacy than women, whereas the effect of gender on self-efficacy was not significant in the CAC profile.

**TABLE 5:** Descriptive statistics (means and standard deviations) corresponding to coping profiles in general self-efficacy.

		Coping profiles			
		
		LACS	HACS	SAC	CAC
		*M* (*SD*)	*M* (*SD*)	*M* (*SD*)	*M* (*SD*)
General self-efficacy	Women	2.73 (0.61)	3.69 (0.55)	3.16 (0.59)	3.62 (0.49)
	Men	3.18 (0.53)	3.96 (0.51)	3.43 (0.49)	3.79 (0.63)
	Total	2.88 (0.60)	3.78 (0.54)	3.22 (0.58)	3.72 (0.58)

*LACS, profile of low approach coping strategies; HACS, profile of high approach coping strategies; SAC, profile with a prevalence of social approach coping strategies; CAC, profile with a prevalence of cognitive approach coping strategies. All measurement scales were from 1 to 5, where the highest scores reflect the highest level of approach coping strategies and general self-efficacy.*

## Discussion

Although previous research has demonstrated the importance of coping strategies and self-efficacy in the prevention of stress, the relationship between these two psychological resources has not been the focus of attention previously in the university context. The main contribution of this study is in the analysis of the relationship between coping strategies and general self-efficacy in university students in light of coping flexibility.

From this person-centered focus, it is assumed that coping strategies are not mutually exclusive categories but instead operate together (Eisenbarth, 2012; Kobylińska and Kusev, 2019), such that their functionality depends on the individuals having a repertoire of strategies available that would allow them to respond specifically to the challenge they have to deal with (Cheng et al., 2014; Siltanen et al., 2019). The results of our study are consistent with this approach, we have identified four profiles of university students which differ in the extent of their flexibility in approach coping with stress. One of the profiles we identified (HACS) has a coping repertoire which combines high levels of positive reappraisal, support seeking, and planning. This is a group of highly flexible students when it comes to coping with problems, bringing together strategies for primary control of stressors (planning and instrumental support seeking) with others aimed at secondary control (positive reappraisal and emotional support seeking). In general, research suggests that when facing problems, the most effective method is to use primary control strategies when the situation is deemed controllable, whereas relying on secondary control strategies is more beneficial when the challenge is perceived as uncontrollable (Zimmer-Gembeck and Skinner, 2016). From this perspective, the HACS profile would be highly adaptive, as the students in this group would have both types of strategy available. Our findings also demonstrated the existence of two profiles of students who displayed lower levels of coping flexibility than the HACS profiles, as their repertoires included high levels of some but not all of the three approach coping strategies we examined. One group was characterized by the combination of high levels of positive reappraisal and planning, with low levels of support seeking (the CAC profile). The other, in contrast, combined high levels of support seeking with low levels of the other two strategies (the SAC profile).

These two profiles are, to a certain extent, opposites, as students in the SAC group exhibited predominantly social coping, prioritizing their sources of support as the routes to find advice and/or emotional consolation about their problematic situations, whereas students in the CAC group preferred to opt for a more cognitive coping (i.e., focus on the positives of the situation and plan how to deal with it) rather than sharing their problems socially. According to this characterization, the students with a SAC profile would have a much smaller repertoire of approach coping strategies, which could indicate excessive instrumental and emotional dependence on their significant social circle when they have to deal with academic and non-academic stressors. Students with a CAC profile would choose to respond to stressors more autonomously, either because of a lack of interpersonal skills to ask for help or because they feel they do not have this social support or because they feel the advantages of seeking help are outweighed by the disadvantages (Scharp and Dorrance Hall, 2019), such as being considered incompetent or weak. Finally, in this study, we identified the existence of a group of students characterized by a low use of positive reappraisal, support seeking, and planning (the LACS profile). Assuming that these three strategies are highly functional in academic contexts (Skinner et al., 2016), the reduced availability of them in this profile would seem to indicate the students’ lack of flexibility to respond adaptively to the various demands of day-to-day university life.

The identification of these four profiles adds to the growing line of work which supports the benefits of analyzing coping with stress in the university context with a person-centered approach (e.g., Cheng, 2001; Kato, 2012; Doron et al., 2014; Freire et al., 2018; Gabrys et al., 2018; González Cabanach et al., 2018; Hasselle et al., 2019). To be specific, the four-profile solution in our study coincides with results from González Cabanach et al. (2018), in a study which also examined flexibility of coping based on the combination of positive reappraisal, support seeking, and planning strategies. This may point to a potential generalization of the profiles identified when the flexibility of approach coping with stress is examined in a university context.

Beyond affirming the existence of student profiles characterized by differences in the flexibility of coping, the objective of our study was to determine whether these groups diverged in their expectations of self-efficacy. In accordance with our hypothesis, the greater the flexibility in approach coping with stress, the higher the students’ levels of general self-efficacy and vice versa. The student profiles that had most flexibility in their coping (HACS and CAC) exhibited notable differences (i.e., large effect sizes) in self-efficacy compared to less flexible profiles (SAC and LACS). Additionally, the SAC profile exhibited moderately higher self-efficacy (i.e., medium effect size) than the LACS profile.

These results could indicate, in line with other studies from the healthcare context (e.g., Haythornthwaite et al., 1998), that flexibility in coping enhances university students’ perception of control over their day-to-day challenges, making them feel better able to handle them. This explanation may be connected with what Hobfoll’s conservation of resources theory (Hobfoll et al., 2018) postulates. According to this theory, individuals who have high levels of personal resources (e.g., a variety of approach coping strategies) participate in an upward spiral of acquisition, development, and preservation of new resources (e.g., self-efficacy). In contrast, scarce resources in the face of a given challenge (e.g., low flexibility in coping) would put the individual into a downward spiral of losing resources (e.g., low self-efficacy) which would make them more vulnerable to stress. In this way, personal resources would act in “convoy” (Holmgreen et al., 2017), one after the other, whether upward or downward. In addition, the fact that we did not find significant differences between the HACS and CAC profiles with regard to general self-efficacy suggests that, in terms of developing generalized self-referential beliefs about personal competency in response to the demands of university life, the combination of cognitive strategies (positive reappraisal and planning) is more important than social strategies (support seeking). This idea is in line with the lower potency that Bandura’s (1997) social cognitive theory ascribes to social sources in making up expectations of self-efficacy. Thus, it is possible that the low availability of cognitive coping resources exhibited by students with the SAC profile would negatively affect their beliefs of competency for dealing with stressors, which would lead them to seek feedback from their sources of support that would give them some degree of self-efficacy, albeit significantly less than students with HACS and CAC profiles, but still somewhat higher than students with the LACS profile.

### Implications of the Results of the Study

University stress is a growing psychosocial concern, both because of its prevalence and because of the negative consequences it can have for the student. Although this scenario highlights the need to implement effective coping interventions in the entire university population, this need is even more pronounced in students who are studying healthcare-related degrees (Saeed et al., 2016), in which stress levels are significantly higher (Heinen et al., 2017; Zeng et al., 2019). In line with that, the results of our study may represent a significant contribution, in that they help increase our understanding of how two important psychological resources, flexibility of approach coping strategies and general self-efficacy, function in the prevention of stress.

To be more specific, our findings allow the identification of those students who, depending on the level of their flexibility in the use of approach coping strategies, are more (LACS and SAC profiles) or less (HACS and CAC profiles) vulnerable with respect to developing their expectations of generalized self-efficacy.

Not only does self-efficacy play an important role in the prevention of university stress (Freire et al., 2019; Schönfeld et al., 2019), it is also one of the most influential factors in the motivational, cognitive, and behavioral responses of the student to the teaching–learning process (Schunk and Pajares, 2010). Consequently, in light of our results, students in the SAC and particularly in the LACS profiles should be the focus of priority intervention in order to enhance flexibility in their repertoire of approach coping strategies as a way of improving their generalized expectations of self-efficacy. In recent years, interventions aimed at improving the coping skills of university students have proliferated. Most of these initiatives have adopted an approach based on cognitive behavioral therapy (Houston et al., 2017), mindfulness (Kang et al., 2009), or a combination of the two (Recabarren et al., 2019). In these programs, students learn to identify the main symptoms associated with stress, as well as the external (environmental demands) and internal (thoughts and emotions) factors that contribute to its appearance. Furthermore, students acquire various primary control (e.g., planning and problem solving) and secondary control (e.g., positive reappraisal and meditation) adaptive coping strategies.

Although these types of interventions have shown their effectiveness both in reducing stress (Regehr et al., 2013; Yusufov et al., 2019) and in increasing self-efficacy (Molla Jafar et al., 2015; Phang et al., 2015), they have limited influence by themselves on the students’ abilities to be flexible in their coping strategies (Cheng and Cheung, 2005). Prior research offers us evidence of the efficacy of focused training to enhance both individuals’ repertoires of strategies and their metacognitive abilities to evaluate and select the best coping strategies in each situation (Cheng et al., 2012).

From this, it would seem that metacognitive self-regulation and executive functioning skills (e.g., planning, organization, emotional management) constitute an important resource for improving students’ abilities to make their repertoires of strategies more flexible, in addition to specific training aimed at increasing their coping strategies (Bettis et al., 2017; de la Fuente et al., 2018a). Some online tools in this area, such as e-Coping with Academic Stress^TM^, have demonstrated good results in the improvement of self-regulating skills (e.g., self-evaluation and decision making) in students when facing potentially stressful situations in the university context (de la Fuente et al., 2018b). These results also have important implications at the classroom level, given that if teachers encourage the development of self-regulation skills in university students, they increase the tendency for students to autonomously use approach coping strategies, such as establishing a plan of action, assessing the positive aspects of the situation, or seeking advice and emotional support from other people (de la Fuente et al., 2020). These self-regulatory skills have also been shown to be effective in increasing students’ self-efficacy beliefs (Cerezo et al., 2019).

### Limitations of the Study and Lines for Future Research

The contributions of this study should be assessed, taking into account the limitations inherent in its design. First, the transversal nature of the study does not allow causal relationships to be established between the variables studied. Therefore, although our results suggest that flexibility in coping with stress influences the generalized expectations of self-efficacy, the causal order between these variables must be examined in the light of more rigorous study designs (e.g., longitudinal studies). A second limitation lies in the composition of the sample, which was dissimilar in terms of gender representation, university year, and degree type. In this study, those three variables were considered as covariates to statistically control their effect, with degree type and gender exhibiting a null effect and a small effect, respectively. However, new studies are needed that would be able to corroborate the extent to which these variables are important, or not, in the configuration of the profiles of coping flexibility and in the relationship between these profiles and self-efficacy. In fact, based on our findings, the levels of general self-efficacy were significantly higher in men (albeit with a small effect size) in all of the coping profiles except the group which had similar levels of representation of both sexes (the CAC profile), where there were no differences. Therefore, in order to make the results more generalizable to the university student population, future studies should use more thorough recruitment procedures that would give more balanced samples in terms of gender, university year, and degree type. In the same vein, future work should consider the extent to which variables not addressed in this study, such as students’ previous academic performance, their socioeconomic status, or their intellectual abilities (e.g., cognitive and attention level), may be relevant in the relationship between stress coping profiles and general self-efficacy in the university context. The fact that all of the participants were recruited from the same university constitutes a third limitation of our study. In order to facilitate generalization of the results, new studies are needed which involve students from other geographical and cultural contexts.

Fourth, the use of self-reports as a data collection method may limit the veracity of the results, since participants may have response biases, ranging from a misunderstanding of the items to social desirability bias (i.e., the tendency of survey respondents to answer questions in a manner that will be viewed favorably by others, even if the survey is anonymous) (Rosenman et al., 2011). These biases may have been increased by the effect of the data collection method used (collective and pencil-and-paper condition). In fact, this type of method can increase the perception of a lack of privacy and confidentiality when other participants are present (van de Looij-Jansen and de Wilde, 2008), encouraging the social desirability response effect and a higher rate of questions not answered, especially with sensitive questions such as those related to mental health (Raat et al., 2007). These and other limitations—for example, data collection costs and data entry errors (Colasante et al., 2019), physical and emotional fatigue of the participants at the time data collection, and absence of a rigorous control over the time taken to complete the questionnaires (Díaz de Rada, 2018)—could be minimized by using computerized administration of questionnaires. Likewise, future studies should corroborate our findings using a combination of methods that include not only questionnaires but also classroom observations and in-depth interviews with the students.

There is another limitation with respect to the questionnaires used, specifically the questionnaire we used to evaluate coping strategies. Although the three strategies evaluated by this instrument (positive reappraisal, support seeking, and planning) are widely used in academic contexts, that does not preclude the possibility of students using other types of strategies. Future research should examine the possible makeup of flexible coping profiles considering other strategies that were not assessed in this study.

Finally, another limitation lies in the operationalization of the concept of coping flexibility. Our results seem to be consistent with the conceptualization of coping flexibility in terms of balanced profiles, according to which the student deploys various strategies at similar levels (Kaluza, 2000). Despite this idea of coping flexibility being widely adopted in the educational field, there are other ways to operationalize this construct (e.g., a broad repertoire or cross-situational variability; for a more precise characterization, see Cheng et al., 2014), which might impede comparison between studies and the generalization of the results.

## Data Availability Statement

The datasets generated for this study are available on request to the corresponding author.

## Ethics Statement

The studies involving human participants were reviewed and approved by the Ethics Committee at the University of A Coruña. The patients/participants provided their written informed consent to participate in this study.

## Author Contributions

CF and MF contributed to the conceptualization, investigation, methodology, writing, and supervision of this study. BR and SR contributed to the investigation, writing, and supervision of this study. AV and JN contributed to the methodology, writing, and supervision of this study.

## Conflict of Interest

The authors declare that the research was conducted in the absence of any commercial or financial relationships that could be construed as a potential conflict of interest.
